# Effect of Salinity and Alkalinity on* Luciobarbus capito* Gill Na^+^/K^+^-ATPase Enzyme Activity, Plasma Ion Concentration, and Osmotic Pressure

**DOI:** 10.1155/2016/4605839

**Published:** 2016-11-17

**Authors:** Longwu Geng, Guangxiang Tong, Haifeng Jiang, Wei Xu

**Affiliations:** Heilongjiang River Fisheries Research Institute, Chinese Academy of Fishery Sciences, Harbin 150070, China

## Abstract

We evaluated the individual and combined effects of salinity and alkalinity on gill Na^+^/K^+^-ATPase enzyme activity, plasma ion concentration, and osmotic pressure in* Luciobarbus capito*. Increasing salinity concentrations (5, 8, 11, and 14 g/L) were associated with an initial increase and then decrease in* L. capito* gill Na^+^/K^+^-ATPase activity. Activity was affected by the difference between internal and external Na^+^ ion concentrations and osmotic pressure (*P* < 0.05). Both plasma ion (Na^+^, K^+^, and Cl^−^) concentration and osmotic pressure increased significantly (*P* < 0.05). An increase in alkalinity (15, 30, 45, and 60 mM) caused a significant increase in plasma K^+^ and urea nitrogen concentrations (*P* < 0.05) but had no effect on either plasma osmotic pressure or gill filament ATPase activity. In the two-factor experiment, the saline-alkaline interaction caused a significant increase in plasma ion (Na^+^, Cl^−^, and urea nitrogen) and osmotic pressure (*P* < 0.05). Variance analysis revealed that salinity, alkalinity, and their interaction significantly affected osmotic pressure, with salinity being most affected, followed by alkalinity, and their interaction. Gill filament ATPase activity increased at first and then decreased; peak values were observed in the orthogonal experiment group at a salinity of 8 g/L and alkalinity of 30 mM.

## 1. Introduction

In China, saline-alkaline water is an important territorial resource. This resource is primarily located in northwestern plateau lakes, in northeastern plain wetlands, and underground in the northern littoral region in China and has an estimated capacity of 539.8 billion m^3^ [1]. The distinguishing features of this water resource include high salinity-alkalinity, poor buffering capacity, and ion imbalance. Coincidentally, these features are also limiting to aquatic animal survival [2]. The availability of water resources is decreasing but demand for aquatic products is increasing [3, 4]. Thus, the use of saline waters will become important to the development of sustainable fisheries. In recent years, China has attempted to breed some fish species in partially saline waters. The species used to date have been restricted to waters with salinity of <5 g L^−1^ and alkalinity <10 mM. However, there is considerable effort being devoted to successfully rearing fish in higher-salinity waters [5–7]. In 2003, China introduced the salt-tolerant fish* Luciobarbus capito* from the Aral Sea in Uzbekistan. This Cyprinidae (Barbinae,* Barbus* genus) is found primarily in the Aral Sea but migrates into the rivers to spawn. In addition to its high salt tolerance, it has a varied diet, has fast growth rate, produces meat that has desirable traits, and is economically important for the region [8].

Gill is the main organ of osmotic regulation in teleosts and chloride cells are the sites of ion transport across gill epithelium [9–11]. ATPases are membrane-bound enzymes responsible for the transport of ions through cell membranes and thus help in regulation of cellular volume, osmotic pressure, and membrane permeability [12]. Salinity tolerance is typically evaluated by measuring gill filament Na^+^/K^+^-ATPase enzyme activity, plasma ion concentration, and osmotic pressure [13–18]. However, because most studies have looked at either salinity [12, 19] or alkalinity [20, 21] alone, it is difficult to evaluate the interactive effect of both factors on aquatic animals. Our objectives were to evaluate the effect of salinity and alkalinity, alone and in combination, on* L. capito* gill Na^+^/K^+^-ATPase activity, plasma ion concentration, and osmotic pressure. Our results can be used to inform the range of saline-alkaline conditions suitable for survival during aquaculture.

## 2. Materials and Methods

### 2.1. Study Species

The experimental fish were 1-year-old, pool-bred fish (40–60 g, 16.57–24.61 cm fork length). The fish were held in a glass-steel circular cylinder (1 m diameter) containing 300 L aerated water (pH 7.51, salinity 0.28‰, total alkalinity 1.39 mM, total hardness 1.10 mM, total phosphorus 0.11 mg/L, total nitrogen 2.95 mg/L, total iron 0.002 mg/L, SO_4_^2−^ 31.19 mg/L, Ca^2+^ 19.81 mg/L, and Mg^2+^ 2.03 mg/L) at room temperature. The fish were acclimated to experimental conditions for 3 d. The analytical reagents (AR) were NaCl and NaHCO_3_.

### 2.2. Experimental Design

First, we evaluated the individual effect of salinity or alkalinity. The fish were divided into four treatment groups for each factor: 5, 8, 11, and 14 g/L NaCl and 15, 30, 45, and 60 mM NaHCO_3_. Second, we evaluated the combined effects of salinity and alkalinity. The fish were divided into 9 treatment groups to test all combinations of salinity (5, 8, and 11 g/L) and alkalinity (15, 30, and 45 mM), based on orthogonal table L_9_ (3^4^) (Table 1). Each treatment group consisted of three replicates with eight specimens per replicate, and the number of the experimental fish was 480. The experiment was performed for 60 days without aeration at 22 ± 0.5°C. A third of the water was changed daily and the corresponding concentration was adjusted with a pre-prepared master mix.

### 2.3. Sample Collection and Testing

After the experiment, the fish were anaesthetized by immersion in Tricaine Methanesulphonate (MS-222) at 200 mg/L. Approximately 1.5–2.0 mL blood was drawn from the caudal vessels, just behind the anal fin, with a 2.5 mL syringe. The samples were centrifuged at 4000 ×g for 10 min and the plasma was collected and frozen at −70°C until required. After collecting the blood, the fish were put into an ice box. The gills were then removed and washed with cold saline water (0.7 g/L), the surface moisture was dried with filter paper, and the gill filament was then accurately weighed to the nearest 0.2 g. The gill tissue was then homogenized in 1.8 mL saline water according to weight and volume and centrifuged at 1200 ×g for 10 min, and the clear supernatant was eluted for testing. Plasma and gill filament test samples were taken from three fish.

Plasma Na^+^, Cl^−^, and K^+^ concentrations were measured by using the detection kits (Nanjing Jiancheng Bioengineering Institute, Nanjing, China). The urea nitrogen was spectrophotometrically measured with Nessler's reagent as the chromogenic reagent according to Lu export method [22]. The activity of Na^+^/K^+^-ATPase was determined by Spectrophotometry using ATP as substrate; thus, 1 *μ*mol inorganic phosphorus generated by ATPase enzyme decomposition is equal to one ATPase enzyme activity unit (*μ*mol Pi/mg prot/h). Coomassie blue staining and calf plasma protein as a standard were used for protein determination [23]. Plasma osmotic pressure was determined using an autofreezing point osmometer (Labsun Technology Development Co. Ltd., Beijing, China).

### 2.4. Statistical Analysis

Count experimental data and significance tests were performed in SPSS 11.5 software (SPSS Inc., Chicago, IL, USA). Each parameter was analyzed separately by one-way ANOVA. Comparisons between different groups and between the treatment groups and control were performed by ANOVA followed by Duncan's* post hoc* test.

## 3. Results

### 3.1. Influence of Salinity-Alkalinity on Plasma Ion Concentration

The plasma ion concentrations for* L. capito* reared at different salinities are shown in Table 2. The concentration of plasma ions K^+^, Na^+^, and Cl^−^ increased significantly with increasing salinity (*P* < 0.05), and the concentration of these three ions peaked when salinity was 14 g/L (234.21 ± 3.69 mM for K^+^, 292.82 ± 7.23 mM for Na^+^, and 0.57 ± 0.04 mM for Cl^−^). Urea nitrogen concentration was significantly higher than that of the control group (*P* < 0.05) at 14 g/L salinity but was not different in the other groups (*P* > 0.05).

The plasma ion concentrations for* L. capito* reared at different alkalinities are shown in Table 3. K^+^ ion concentration in the experimental groups was significantly higher than in the control group. Na^+^ and Cl^−^ ion concentrations were not significantly affected by changes in alkalinity (*P* > 0.05). Urea nitrogen concentration in the experimental groups was significantly higher than in the control group (*P* < 0.05), and urea nitrogen concentration increased gradually with increasing alkalinity.

The single-factor experiment results revealed that salinity significantly influenced Na^+^, K^+^, and Cl^−^ plasma concentrations but had only a small influence on urea nitrogen concentration. Alkalinity had little effect on plasma Na^+^ and Cl^−^ concentrations but significantly affected K^+^ and urea nitrogen concentrations (*P* < 0.05).

The* L. capito* plasma ion concentrations in the saline-alkaline orthogonal test are shown in Table 4. With increasing salinity and alkalinity, the concentration of plasma ions Na^+^, K^+^, Cl^−^, and urea nitrogen increased to different degrees, suggesting that salinity and alkalinity have an interactive effect on the concentrations of these four plasma ions. The variance analysis revealed that salinity and the saline-alkaline interaction significantly affected K^+^ ion concentrations (*P* < 0.05) but that alkalinity had no significant effect (*P* > 0.05). The effect of salinity on Na^+^ ion concentration was most significant (*P* < 0.05), followed by alkalinity, and their interaction had no significant effect (*P* > 0.05). Both Cl^−^ ion and urea nitrogen concentrations were significantly affected by salinity, alkalinity, and their interaction (*P* < 0.05).

### 3.2. Influence of Salinity-Alkalinity on Plasma Osmotic Pressure

**Figure 1:**
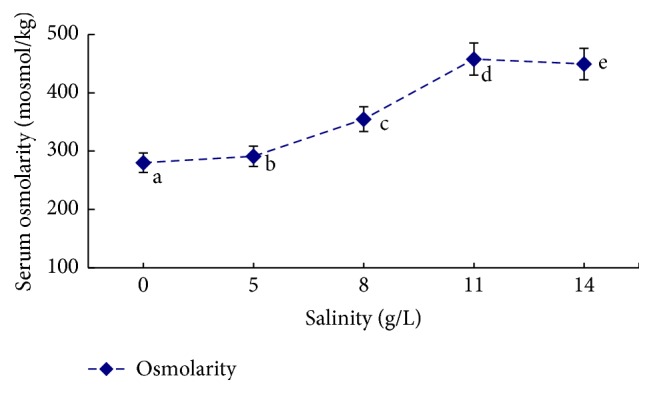
The influence of different salinity conditions on plasma osmolarity in* L. capito*.

**Figure 2 fig2:**
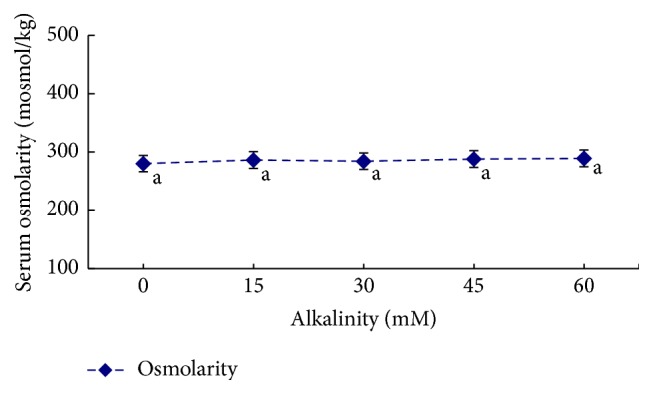
The influence of different alkalinity conditions on plasma osmolarity in* L. capito*.

**Figure 3:**
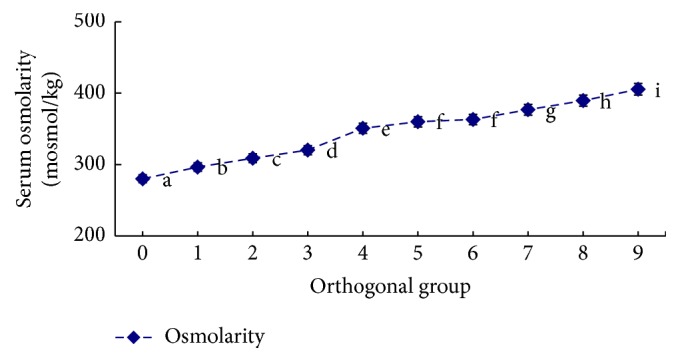
The influence of different saline-alkaline conditions on plasma osmolarity in* L. capito*.

The influence of salinity, alkalinity, and saline-alkaline orthogonal function on plasma osmotic pressure is shown in Table 2 and Figures 1 [Fig fig2]–3. In the single-factor experiment, plasma osmotic pressure significantly increased with increasing salinity (*P* < 0.05) (Figure 1). Increasing alkalinity had no significant effect on plasma osmotic pressure (*P* > 0.05) (Figure 2). In the orthogonal experiment, plasma osmotic pressure gradually increased with increasing salinity and alkalinity concentrations (*P* < 0.05) (Figure 3). The variance analysis revealed that salinity, alkalinity, and their interaction significantly affected osmotic pressure; salinity had the most significant effect, followed by alkalinity, with their interaction being the smallest. The regression equation for plasma osmotic pressure (*Y*), salinity (*S*), and alkalinity (*A*) is *Y* = 260.148 + 10.726*S* + 0.306*A* (*R* ^2^ = 0.904).

### 3.3. Influence of Salinity-Alkalinity on Gill Na^+^/K^+^-ATPase Enzyme Activity

**Figure 4:**
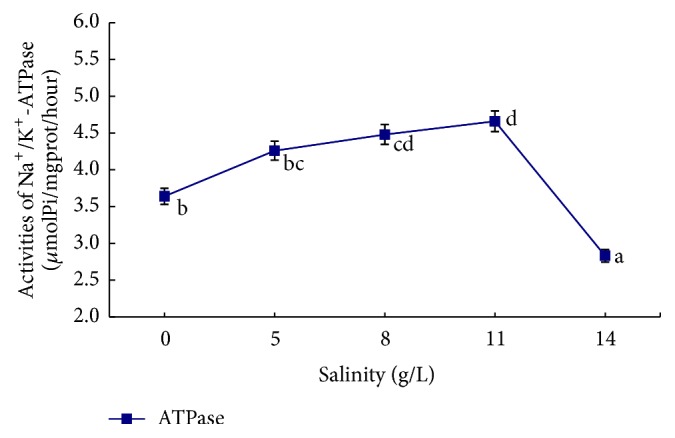
The influence of different salinity conditions on Na^+^/K^+^-ATPase activities in* L. capito*.

**Figure 5 fig5:**
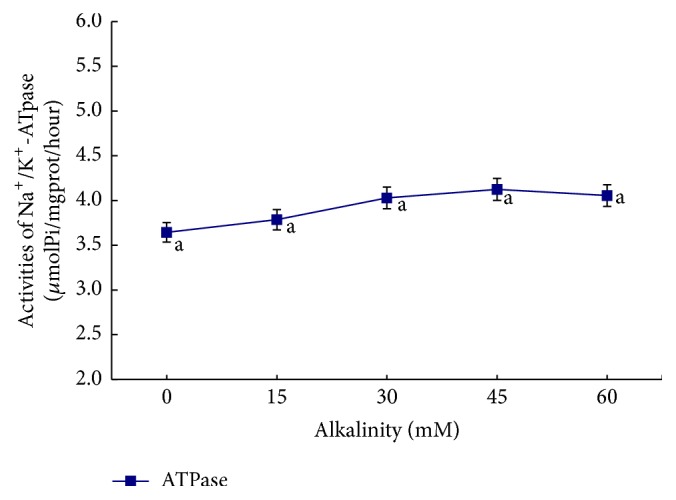
The influence of different alkalinity conditions on Na^+^/K^+^-ATPase activities in* L. capito*.

**Figure 6:**
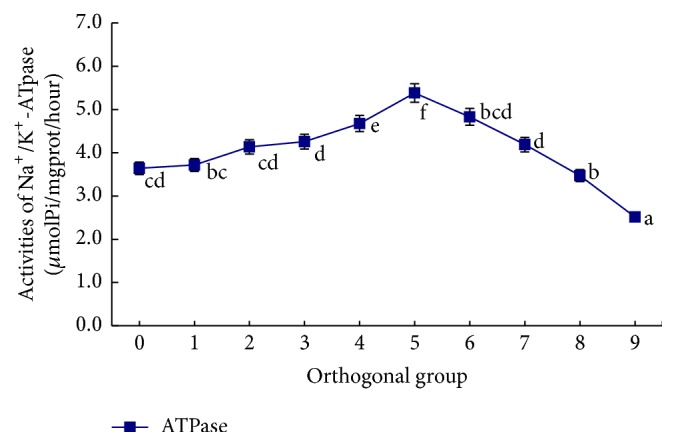
The influence of different saline-alkaline conditions on Na^+^/K^+^-ATPase activities in* L. capito*.

The influence of salinity, alkalinity, and their interaction on gill filament Na^+^/K^+^-ATPase enzyme activity is shown in Figures 4 [Fig fig5]–6. In the single-factor experiment, gill filament Na^+^/K^+^-ATPase enzyme activity significantly increased when salinity was between 5 and 11 g/L (*P* < 0.05), whereas it was significantly lower at salinity >11 g/L (*P* < 0.05) (Figure 4). The influence of increasing alkalinity on gill filament ATPase enzyme activity was not significant (*P* > 0.05) (Figure 5). In the orthogonal experiment, gill filament ATP enzyme activity increased at first and then decreased with the interactive effects of increasing salinity and alkalinity. ATPase enzyme activity peaked in group 5 (salinity 8 g/L and alkalinity 30 mM) and was lowest in group 9 (salinity 11 g/L and alkalinity 45 mM) (Figure 6).

For the single-factor salinity experiment, the difference between gill filament Na^+^/K^+^-ATPase enzyme activity and Na^+^ ion concentration in the water, plasma, and water osmotic pressure and the difference between plasma Na^+^ concentration and Na^+^ concentration in the water are shown in Table 5. In the water, Na^+^ concentration and ATPase enzyme activity were positively correlated but the degree of correlation was not high. Differences in osmotic pressure and Na^+^ concentration were strongly positively correlated with ATPase enzyme activity. ATPase enzyme activity gradually increased with increasing osmotic pressure differences and was highest (4.66 ± 0.07) when the osmotic pressure difference was at its peak (97.17 ± 4.34). The results of the single-factor salinity experiment suggest that differences in plasma osmotic pressure and plasma Na^+^ concentration are important factors affecting gill filament Na^+^/K^+^-ATPase enzyme activity in* L. capito* both* in vitro* and* in vivo*.

## 4. Discussion

### 4.1. Changes in* Luciobarbus capito* Gill Na^+^/K^+^-ATPase Enzyme Activity

All freshwater and saltwater fish exhibit Na^+^/K^+^-ATPase enzyme activity in the gill epithelia. The main function of this is to maintain ion permeability in the cytoplasmic membrane, relative stability of various ion concentrations in the intracellular environment, and osmotic pressure balance between the intracellular and external environments [24–26]. Gill filament ATPase enzyme activity adjusts accordingly when ion concentrations in the external environment change. Generally, ATPase enzyme activity is positively correlated with the external salinity concentration [13, 18, 27, 28]. Based on our single-factor experimental results, changes in gill filament ATPase enzyme activity follow the above-mentioned rule when salinity is <11 g/L. ATPase enzyme activity increased with increasing salinity in the water. This increased the permeability of the gill epithelium membrane, accelerated the discharge of Na^+^ and Cl^−^ ions, and discharged excess salt ions out of the body. This maintained plasma ion concentration at a level required for physical activity, thus ensuring normal activity and energy metabolism [18].

When salinity was >11 g/L and under high salinity stimulus, gill filament Na^+^/K^+^-ATPase enzyme activity in* L. capito* decreased. Some researchers have reported a similar phenomenon in* Chanos chanos* [29]; ATPase enzyme activity in juvenile cobia was lower at 20 g/L salinity than at 10 g/L. Xu et al. have suggested that salinity is not the only factor affecting juvenile cobia gill ATPase activity but that it may be associated with a different regulatory mechanism [15]. Our salinity experimental results revealed that the influence of differences in osmotic pressure or Na^+^ ion concentration* in vivo* and* in vitro* on gill filament ATPase enzyme activity in* L. capito* was significant and did not increase with increasing salinity in the water. The fish improve ATPase enzyme activity and discharged excess salt ions from the body when there was a large difference between plasma osmotic pressure* in vivo* and water osmotic pressure* in vitro*. In contrast, the ATPase enzyme activity was decreased, when the difference was smaller.

In the saline-alkaline orthogonal experiment, the overall trend of changes in gill filament Na^+^/K^+^-ATPase enzyme activity was similar to that for salinity, but peak ATPase enzyme activity was higher than that in the single-factor salinity experiment. The salinity concentration corresponding to peak ATPase activity was lower than in the single-factor, suggesting that the saline-alkaline interaction has a greater influence on ATPase enzyme activity than salinity alone. At certain concentrations, alkalinity impedes ion exchange in gill tissue exposed to a salt solution. The gill tissue needs to maintain a smooth ion channel; therefore, gill filament ATPase enzyme activity must increase. A similar phenomenon was observed in our experiment; alkalinity stimulus increased mucus secretion from the fish body. Much of the mucus condensed on the surface of the gill filament, directly affecting normal breathing and other gill tissue functions.

### 4.2. Osmotic Pressure Regulation in* Luciobarbus capito*

The plasma osmotic pressure of stenohaline marine bony fishes is 370–480 mOsm/kg, but that of stenohaline freshwater bony fishes is 260–330 mOsmol/kg [15]. The plasma osmotic pressure range in* L. capito* is 280–457 mOsm/kg. Thus,* L. capito* is considered a euryhaline fish because its plasma osmotic pressure range is between those of marine and freshwater bony fishes. Typically, when a euryhaline bony fish enters high salinity water, osmotic pressure regulation occurs in two stages. First, as a result of passive water loss, the osmotic pressure increases because of a relative increase in plasma ion concentrations. Second, gill filament Na^+^/K^+^-ATPase enzyme activity increases and the ion discharge mechanism is activated, which causes osmotic pressure to gradually decrease and finally level off [13, 15, 30–32]. Plasma osmotic pressure in* L. capito* initially increased with increasing salinity in the water, as normal, yet it did not gradually decrease and level off. Thus, the change in plasma osmotic pressure in* L. capito* is a response to the external hypertonic environment. Whether this is a unique reaction of this species to saline-alkaline waters requires further study.

Urea nitrogen content in* L. capito* changed under different saline-alkaline concentrations, suggesting that plasma urea also participates in osmotic pressure regulation. However, the regulation of urea to osmotic pressure was only significantly affected by alkalinity and saline-alkaline mixed water. In the single-factor salinity experiment, the regulation of urea to osmotic pressure was lower. Urea is a key factor in maintaining the balance between water and salt in elasmobranchs. When urea content is higher in the blood, the blood osmotic pressure is higher than the surrounding environment and close to isotonic, thus reducing moisture loss [26]. Alkalinity is the limiting factor for aquatic animal survival and growth in saline-alkaline waters; most euryhaline fishes find it difficult to overcome alkalinity putting them at a disadvantage in saline-alkaline waters [33]. Our results show that, in alkaline water environments, urea supplements other ions regulating osmotic pressure, enhances the osmotic regulation ability of* L. capito* in saline-alkaline waters, and thus improves its tolerance for saline-alkaline waters.

## 5. Conclusions

*Luciobarbus capito* gill Na^+^/K^+^-ATPase activity, plasma ion (Na^+^, K^+^, and Cl^−^) concentration, and osmotic pressure were increased at first and then decreased with increasing salinity.* L. capito* plasma K^+^ and urea nitrogen concentrations increased with increasing alkalinity. All these concentrations gradually increased with increasing salinity and alkalinity concentrations in the orthogonal experiment.

## Figures and Tables

**Table 1:** The orthogonal test of salinity and alkalinity.

Experience group number	Treatment level	
	NaCl (g/L)	NaHCO_3_ (mM)
0	0	0
1	5	15
2	5	30
3	5	45
4	8	15
5	8	30
6	8	45
7	11	15
8	11	30
9	11	45

**Table 2:** Plasma Na^+^, Cl^−^, and K^+^ concentration, ammonia content, and osmolarity in *L. capito *at different salinity conditions.

NaCl (g/L)	Na^+^ (mM)	Cl^−^ (mM)	K^+^ (mM)	BUN (mM)	Osmolarity (mosmol/kg)
0	153.77 ± 8.72^a^	105.81 ± 3.85^a^	0.35 ± 0.05^a^	1.36 ± 0.08^a^	280.10 ± 4.76^a^
5	161.84 ± 4.44^b^	119.17 ± 3.18^b^	0.49 ± 0.06^b^	1.43 ± 0.11^a^	291.02 ± 2.57^b^
8	216.41 ± 8.82^c^	174.85 ± 5.75^c^	0.52 ± 0.03^b^	1.47 ± 0.10^a^	354.95 ± 4.83^c^
11	276.11 ± 5.99^d^	232.18 ± 4.23^d^	0.53 ± 0.04^b^	1.53 ± 0.06^a^	457.81 ± 2.79^d^
14	292.82 ± 7.23^e^	234.21 ± 3.69^d^	0.57 ± 0.04^bc^	1.63 ± 0.12^b^	449.32 ± 4.81^e^

Different letters in the columns indicate significant differences between variables evaluated (*P* < 0.05).

**Table 3:** Plasma Na^+^, Cl^−^, and K^+^ concentration, ammonia content, and osmolarity in *L. capito *at different alkalinity conditions.

NaHCO_3_ (mM)	Na^+^ (mM)	Cl^−^ (mM)	K^+^ (mM)	BUN (mM)	Osmolarity (mosmol/kg)
0	153.77 ± 8.72^a^	105.81 ± 3.85^a^	0.35 ± 0.05^a^	1.36 ± 0.08^a^	280.10 ± 4.76^a^
15	161.22 ± 5.87^a^	109.61 ± 7.33^a^	0.58 ± 0.06^b^	5.06 ± 0.15^c^	286.27 ± 5.63^a^
30	153.30 ± 6.89^a^	105.21 ± 5.71^a^	0.61 ± 0.03^b^	5.04 ± 0.24^c^	284.16 ± 3.11^a^
45	152.21 ± 6.57^a^	104.37 ± 5.55^a^	0.64 ± 0.04^b^	4.87 ± 0.19^c^	287.88 ± 4.85^a^
60	151.78 ± 6.03^a^	103.00 ± 4.21^a^	0.65 ± 0.05^b^	4.57 ± 0.38^bc^	288.98 ± 5.62^a^

Different letters in the columns indicate significant differences between variables evaluated (*P* < 0.05).

**Table 4:** Plasma Na^+^, Cl^−^, and K^+^ concentration, ammonia content, and osmolarity in *L.capito* at different saline-alkaline conditions.

Group number (salinity × alkalinity)	Na^+^ (mM)	Cl^−^ (mM)	K^+^ (mM)	BUN (mM)	Osmolarity (mosmol/kg)
0 × 0	153.77 ± 8.72^a^	105.81 ± 3.85^a^	0.35 ± 0.05^a^	1.36 ± 0.08^a^	280.10 ± 4.76^a^
5 × 15	155.68 ± 3.84^a^	113.33 ± 1.85^b^	0.32 ± 0.05^a^	1.30 ± 0.08^a^	296.66 ± 3.69^b^
5 × 30	164.77 ± 1.88^b^	116.70 ± 2.88^b^	0.39 ± 0.04^a^	1.76 ± 0.05^b^	308.84 ± 2.16^c^
5 × 45	169.91 ± 2.49^b^	118.40 ± 2.62^b^	0.39 ± 0.05^a^	1.71 ± 0.03^b^	320.21 ± 4.31^d^
8 × 15	180.40 ± 3.70^c^	118.00 ± 2.81^b^	0.43 ± 0.05^a^	1.79 ± 0.03^b^	350.91 ± 1.41^e^
8 × 30	189.20 ± 2.31^c^	144.50 ± 2.26^c^	0.49 ± 0.07^b^	1.71 ± 0.04^b^	359.94 ± 5.46^f^
8 × 45	191.25 ± 3.61^d^	156.33 ± 2.23^d^	0.55 ± 0.06^b^	1.87 ± 0.03^c^	363.15 ± 3.12^f^
11 × 15	195.68 ± 2.66^d^	173.71 ± 3.19^f^	0.64 ± 0.03^c^	3.31 ± 0.06^e^	376.81 ± 4.67^g^
11 × 30	205.06 ± 3.69^e^	177.87 ± 2.75^f^	0.56 ± 0.04^b^	3.19 ± 0.06^d^	389.65 ± 3.96^h^
11 × 45	209.64 ± 2.25^e^	169.23 ± 1.50^e^	0.54 ± 0.06^b^	3.38 ± 0.09^e^	405.52 ± 4.80^i^
ANOVA	*S* > *A*,	*S* > *A* > *S* × *A*	*S* > *S* × *A*,	*S* > *A* > *S* × *A*	
	*S* × *A* not significant		*A* not significant		

*S*, salinity effect. *A*, alkalinity effect. *S* × *A*, interaction of salinity and alkalinity. Different letters in the columns indicate significant differences between variables evaluated (*P* < 0.05).

**Table 5:** The correlation of water Na^+^ concentration, serum osmosis, and Na^+^ concentration difference to the Na^+^/K^+^-ATPase of* L. capito* at different salinity conditions.

NaCl (g/L)	Na^+^ concentration (mM)	Serum osmosis difference (mosmol/kg)	Na^+^ concentration difference (mM)	Na^+^/K^+^-ATPase (*μ*molPi/mgprot/hour)
5	90.47 ± 5.78	92.46 ± 5.53	71.38 ± 5.20	4.26 ± 0.13
8	139.56 ± 7.31	94.41 ± 10.41	76.85 ± 15.82	4.48 ± 0.13
11	194.29 ± 3.90	97.17 ± 4.34	81.81 ± 4.91	4.66 ± 0.07
14	243.92 ± 3.25	12.87 ± 3.18	48.90 ± 4.98	2.83 ± 0.20
Regression equation	*Y* = 3.889 + 0.004*X* (*R* ^2^ = 0.684)	*Y* = 2.599 + 0.020*X* (*R* ^2^ = 0.922)	*Y* = 1.319 + 0.038*X* (*R* ^2^ = 0.872)	—
